# What Is eHealth (4): A Scoping Exercise to Map the Field

**DOI:** 10.2196/jmir.7.1.e9

**Published:** 2005-03-31

**Authors:** Claudia Pagliari, David Sloan, Peter Gregor, Frank Sullivan, Don Detmer, James P Kahan, Wija Oortwijn, Steve MacGillivray

**Affiliations:** ^5^RAND EuropeLeidenThe Netherlands; ^4^Department of Health Evaluation SciencesUniversity of VirginiaCharlottesville VAUSA; ^3^Tayside Centre for Health InformaticsUniversity of DundeeDundeeUnited Kingdom; ^2^Division of Applied ComputingUniversity of DundeeDundeeUnited Kingdom; ^1^Division of Clinical and Community Health Sciences (General Practice Section)University of EdinburghEdinburghUnited Kingdom

**Keywords:** eHealth, Internet, telemedicine, medical informatics

## Abstract

**Background:**

Lack of consensus on the meaning of eHealth has led to uncertainty among academics, policymakers, providers and consumers. This project was commissioned in light of the rising profile of eHealth on the international policy agenda and the emerging UK National Programme for Information Technology (now called Connecting for Health) and related developments in the UK National Health Service.

**Objectives:**

To map the emergence and scope of eHealth as a topic and to identify its place within the wider health informatics field, as part of a larger review of research and expert analysis pertaining to current evidence, best practice and future trends.

**Methods:**

Multiple databases of scientific abstracts were explored in a nonsystematic fashion to assess the presence of eHealth or conceptually related terms within their taxonomies, to identify journals in which articles explicitly referring to eHealth are contained and the topics covered, and to identify published definitions of the concept. The databases were Medline (PubMed), the Cumulative Index of Nursing and Allied Health Literature (CINAHL), the Science Citation Index (SCI), the Social Science Citation Index (SSCI), the Cochrane Database (including Dare, Central, NHS Economic Evaluation Database [NHS EED], Health Technology Assessment [HTA] database, NHS EED bibliographic) and ISTP (now known as ISI proceedings).We used the search query, “Ehealth OR e-health OR e*health”. The timeframe searched was 1997-2003, although some analyses contain data emerging subsequent to this period. This was supplemented by iterative searches of Web-based sources, such as commercial and policy reports, research commissioning programmes and electronic news pages. Definitions extracted from both searches were thematically analyzed and compared in order to assess conceptual heterogeneity.

**Results:**

The term eHealth only came into use in the year 2000, but has since become widely prevalent. The scope of the topic was not immediately discernable from that of the wider health informatics field, for which over 320000 publications are listed in Medline alone, and it is not explicitly represented within the existing Medical Subject Headings (MeSH) taxonomy. Applying eHealth as narrative search term to multiple databases yielded 387 relevant articles, distributed across 154 different journals, most commonly related to information technology and telemedicine, but extending to such areas as law. Most eHealth articles are represented on Medline. Definitions of eHealth vary with respect to the functions, stakeholders, contexts and theoretical issues targeted. Most encompass a broad range of medical informatics applications either specified (eg, decision support, consumer health information) or presented in more general terms (eg, to manage, arrange or deliver health care). However the majority emphasize the communicative functions of eHealth and specify the use of networked digital technologies, primarily the Internet, thus differentiating eHealth from the field of medical informatics. While some definitions explicitly target health professionals or patients, most encompass applications for all stakeholder groups. The nature of the scientific and broader literature pertaining to eHealth closely reflects these conceptualizations.

**Conclusions:**

We surmise that the field – as it stands today – may be characterized by the global definitions suggested by Eysenbach and Eng.

## Introduction

The application of information and communications technology (ICT) in health care has grown exponentially over the last 15 years and its potential to improve effectiveness and efficiency has been recognized by governments worldwide [1]. National strategies aimed at developing health information infrastructures and “infostructures” are emerging across North America, Australia, Europe and elsewhere [2-5]. These are united by a vision to improve the safety, quality and efficiency of patient care by enabling access to electronic health records and by supporting clinical practice, service management, research and policy though availability of appropriate evidence and data. In addition, these strategies emphasize the importance of standards and policies for ensuring interoperability and data security, and many incorporate a commitment to facilitate consumer empowerment and patient self-care through provision of electronic information and/or telemedicine facilities. In the United Kingdom, these principles are reflected in the National Information Strategy for Health and are being addressed via the UK National Programme for Information Technology (NPfIT, now called Connecting for Health) and related initiatives [6,7].

While such initiatives have been taking place, the focus of health care information technology (IT) has been changing, from an emphasis on hardware, systems architectures and databases, to innovative uses of technology for facilitating communication and decision making, coupled with a growing recognition of the importance of human and organizational factors. At the same time, Internet technologies have become increasingly pervasive. In parallel, the language of health care IT has been changing, and references to the concept of eHealth have proliferated in international health policy, management and research arenas. Despite the clear interest in and apparent marketability of eHealth, it was not evident, at the time this research was commissioned, what exactly was meant by the term. It had been variously used as a synonym for health informatics, telemedicine, consumer health informatics and e-business, as well as more specific technological applications, but no consensus existed on its conceptual scope and it was unclear whether it indeed represented a new concept, or simply a linguistic change. An international call for definitions of eHealth posted in 2001 failed to generate any published responses and the call was updated in June 2004, suggesting that this is still a grey area [8,9].

In view of these uncertainties, it was considered important by the UK National Health Service (NHS) Research and Development Programme to define eHealth and to assess its scope and value for the future of health care, in particular to synthesize the available evidence relating to its potential impact, likely trajectory, and implications for service development and organization. The current paper reports descriptive work to profile and define the field, which was conducted independently of, but complements, the systematic review of definitions of eHealth provided elsewhere in this volume [10]. This work produced a framework for locating evidence on the effectiveness, promise and challenges of eHealth, as well as recommendations for future research, which are reported elsewhere [11].

Potential areas of eHealth considered at the outset of the project are shown in Table 1. This was derived by group discussion among the research team, utilizing team members' a priori knowledge of topics and issues in medical informatics (drawing on backgrounds in health care research, practice, policy, and computing), key eHealth discussion papers, and the results of a preliminary Medline search suggesting that eHealth is closer to the emerging area of health informatics than to medical informatics as a whole. While it was established that eHealth is about the use of information technology to facilitate patient and citizen health care or service delivery, rather than technology per se, uncertainty remained about what specific topics or issues, among those shown, fall within the scope of, or have relevance to, the concept.

It was recognized that in order to fully explore the area, multiple sources of information would need to be examined. While identifying the scope of eHealth research was a crucial objective, the published research literature presents a filtered record of activity and thinking and, given the fast-moving pace of the field and its importance beyond academia, nonresearch sources are likely to yield rich information about the current status of eHealth and future trends. For this reason we conducted two parallel, large scale reviews—one focusing on the medical and related scientific literature and the other drawing on alternative sources available via the World Wide Web, including independent scoping exercises (of which there have been several), policy documents and technology reports. The results of these exercises were converged in order to derive a conceptual map and are considered together in this report.

**Table 1:** Potential eHealth areas and issues considered at the outset of the project

**Whatissues currently dominate eHealth?** **What is going on in eHealth?**			**What emerging technologies are likely to impact on health care?**	**How does research inform eHealth?**	**How do developments in eHealth inform research?**
**Professional Clinical Informatics** - Decision aids for practitioners (eg, prompts, reminders, care pathways, guidelines) - Clinical management tools (eg, electronic health records [EHRs/EPRs], audit tools) - Educational aids (guidelines, medical teaching) - Electronic clinical communications tools (eg, e-referral, e-booking, e-discharge correspondence, clinical email/second opinion, laboratory test requesting/results reporting, e-shared care) - Electronic networks (NHS-Net and disease-specific clinical networking systems) - Discipline/disease-specific tools (eg, diabetes informatics) - Telemedicine applications (for interprofessional communication, patient communication and remote consultation) - Subfields eg, nursing & primary care informatics)	**Electronic Patient/Health Records (EPR, EHR)** - Electronic medical records. Record linkage. The Universal Patient Indicator. Databases and population registers. - Achieving multiprofessional access. Technical and ethical issues. - Data protection/security issues - Patient access and control - Integration with other services (eg, social work, police) - Clinical coding issues (terminologies, etc) **Healthcare Business Management** - Billing and tracking systems - Audit & quality assessment systems	**Consumer Health Informatics** - Decision aids for patients facing difficult choices (eg, genetic screening) - Information on the web and/or digital TV (public information and educational tools for specific clinical groups) - Clinician-patient communication tools: 1. Remote: Clinical email and web-based messaging systems for consultation, disease monitoring, service-oriented tasks (eg, appointment booking, prescription reordering). 2. Proximal: Shared decision making tools, informed consent aids 3. Mixed: On-line screening tools (eg, for depression) and therapeutic interventions (eg, cognitive behaviour therapy) - Access and equity issues (data protection issues, the Digital Divide) - Quality issues for health information on the net - “virtual” health communities	**New Technologies** - Satellite communications (eg, for remote medicine ) - Wireless networks (eg, within hospitals, across geographical areas) - Palmtop technologies (for information, for records) - New mobile telephones - Digital TV (for disseminating health information & communicating with patients) - The WWW and it's applications for health (issues: quality control, confidentiality, access) NHS-Direct etc. - Virtual reality (eg, remote/transcontinental surgery) - Nanotechnology - Intersection of bioinformatics and health informatics.	**Research Input** - Development - Need for user involvement in product conception, design and testing. Iterative development. Needs assessment, accessibility and usability research. Multi-faceted expertise required. - Implementation – Understanding people and organizational factors eg, system acceptability, resistance to change etc. Use of tailored implementation strategies. - Innovative methods for mapping functional and technology needs eg, place of systems in the organization - Knowledge management, systems approaches, communication networks models, organizational development to map pathways. - Evaluation Formative, as above, also: Outcome assessment to establish impact of new systems on clinical outcomes, processes and costs. )	**Research Outcomes** - Potential of electronic databases such as population registers for epidemiological research. - Research into the impact or use of informatics tools suggests appropriate and cost-effective priorities for policymakers. - Areas of cross-over (eg, bioinformatics)

## Methods

### Assessing the Taxonomic Structure of Research Databases and the Presence of eHealth

In the formative stage of the project, we explored the subject taxonomies, or thesauri, of multiple databases of abstracts in order to identify high-level subject headings which could be used to profile the volume and content of the medical informatics literature and to construct searches for pertinent evidence. In the case of Medline the thesaurus containing a hierarchical controlled vocabulary is referred to as Medical Subject Headings, or MeSH (see below). As part of this we sought to assess whether eHealth was explicitly represented within these thesauri. A further objective was to determine the ontological structure of the databases in relation to medical informatics and eHealth and the implied relationships between alternative subfields.

The databases examined were Medline (PubMed), the Cumulative Index of Nursing and Allied Health Literature (CINAHL), the Science Citation Index (SCI), the Social Science Citation Index (SSCI), the Cochrane Library Database (including Dare, Central, NHS Economic Evaluation Database [NHS EED], Health Technology Assessment [HTA] database, NHS EED bibliographic) and Index to Scientific and Technical Proceedings (ISTP, now known as ISI proceedings), all of which predate the targeted search period.

### Exploring the Composition of the Medical Informatics Literature Using the Existing MeSH Thesaurus

MeSH has been developed (and is constantly updated by) the US National Library of Medicine. It consists of sets of terms naming descriptors in a hierarchical structure that permits searching at various levels of specificity. At the most general level of the hierarchical structure are very broad headings such as *Anatomy* or *Information Science*. More specific headings are found at more narrow levels of the eleven-level hierarchy, such as *Ankle* or *Medical Informatics*. There are 22568 descriptors in MeSH.

Historical trends in the literature indexed by the individual Medline MeSH terms subsumed within the broad *Medical Informatics* category were assessed for the period 1987 to 2003, and part way through 2004. Individual MeSH definitions were examined to assess the range and nature of the topics covered and to clarify which are most clearly related to common conceptions of eHealth (eg, specific applications of information technology (IT) to health care versus technical issues). The number of publications in Medline was profiled by year, as was the type of publication, subject to the limitations of the Medline categorization scheme (Randomized Controlled Trial/Controlled Trial/Meta-analysis/Review). In addition, the MesH tree was compared with an expert-derived taxonomy from the International Medical Informatics Association (IMIA) in order to assess its coverage of key areas and its merits as a means of identifying appropriate literature.

### Using eHealth as a Search Term

Applying eHealth as free-text search term to multiple databases offered a “grounded” method of defining the field, as represented in the research literature. In order to identify publications specificially relating to eHealth and to place the concept within the wider medical informatics literature, all the databases described previously were searched for the presence of the word *eHealth* or its variants in the title or abstract for the period January 1, 1997 to December 31, 2003 (search string: Ehealth OR e-health OR e*health). Results were organized to show the number of articles arising each year, the journals in which they appeared, and the range of topics covered.

### Profiling the Literature From Wider Web-Based Sources

Mixed methods were used to (*a*) identify current commentary and analysis relating to the emergence, nature, scope and potential of eHealth, and (*b*) locate evidence and opinions on general trends in technology and technology adoption with direct or indirect relevance to eHealth now or in the future. Relevant terms (including *e health, e-health, ehealth, healthcare information technology* and *healthcare computing*) were applied, singly and in combinations, to the Google search engine, which indexes over 8 billion URLs and ranks results by relevance and link popularity. In addition, websites previously identified as being likely to contain information relevant to eHealth were visited directly and scrutinized for pertinent information. In some cases, this was guided by the results of preliminary Google searches or by following up leads suggested in documents found earlier on, while in others it was guided by the existing knowledge of team members. As the searches were predominantly opportunistic and iterative in nature, it is inappropriate to try to document them exhaustively; however, the following types of information were targeted:

- previous exercises to map, scope or define eHealth;
- white papers, technical reports, predictions and early research reports on aspects of technology in health care, eHealth related policy, evaluation and trends, from the United Kingdom, Europe and beyond;
- funding programmes for eHealth- and/or health-and-technology - focused research and development;
- relevant articles from computing and information science-focused academic publications;
- eHealth and health technology-focused websites, web logs and online journals, online ehealth news feeds, email discussion groups and email newsletters;
- online sources with a focus on human-computer interaction, usability and accessibility, with specific attention on health care issues;
- technology-oriented news websites profiling general and health-related trends and developments;
- online studies, reports and statistical surveys relating to general technology take-up; consumer purchasing trends; attitudes and strategies of consumers and clinicians towards adoption of technology in general and for health care-focused tasks in particular; evaluation of the effectiveness of technological innovation, in the health care sector and beyond.

Given the increasing online availability of refereed academic literature there was inevitably some overlap between the information identified by the two searches.

### Aggregating and Analyzing Definitions of eHealth

Scientific abstracts identified using the key word search were examined in order to assess the presence of definitions. While hand searching of full text articles was not a primary objective, this was done where easy Web-based access to this information was available. In the case of Web-based reports or commentary the definition was extracted from the page in which it appeared or was quoted. In both cases the initial extraction was performed by one research fellow and the results checked for inclusion eligibility by a second investigator. Our aim was not to perform an exhaustive and systematic review of definitions (because of time constraints) but to aggregate those appearing most easily and commonly in the research and wider arenas, as a means of supplementing our wider scoping study. The aggregated definitions were then analyzed thematically in order to assess the applications, stakeholders, contexts and theoretical perspectives targeted, so that the heterogeneity of conceptualizations could be determined. They were also considered with reference to the perspectives of the defining individual or organization and associated clarifications within the source document.

## Results

### Assessing the Taxonomic Structure of Research Databases and the Presence of eHealth

Of the databases of scientific abstracts consulted, only Medline has a comprehensive hierarchical taxonomy of descriptors for the broad field of medical informatics. This part of the MeSH tree is shown in Figure 1. Medical informatics is also represented on CINAHL; however the subtree is relatively shallow and undifferentiated, forming only a small branch of the higher *Information Science* category, with many potentially relevant areas subsumed within other branches.

That eHealth has yet to be explicitly included among these thesauri, indicates the relative youth of the topic and the lack of an agreed conceptual definition. The literature relevant to eHealth is thus distributed among a range of existing MeSH fields.

The Medline MeSH structure for *Medical Informatics* contains 3 main subbranches: *Public Health Informatics*, *Medical Informatics Computing*, and *Medical Informatics Applications*. Examining the definitions of these and their lower order MeSH descriptors indicates that the *Medical Informatics Applications* tree encompasses the greatest number of component categories relevant to eHealth, taken broadly as the use of information and communication technologies to facilitate health care. For example, it subsumes the lower-order categories of *Decision Making*, *Computer Assisted* (which subsumes *Computer Assisted Therapy and Diagnosis*, among others); *Information Systems* (electronic information systems, networks, clinical decision support) and *Information Storage and Retrieval* (databases, laboratory information systems, etc). In contrast, *Medical Informatics Computing* is mainly characterized by an emphasis on systems and hardware, although it does contain MeSH descriptors relevant to eHealth — most importantly *Internet*, which may appear in eHealth publications as a specific technology or an application of technology. *Public Health Informatics* is concerned with the application of information and computer sciences to public health practice, research, and learning. Although this potentially encompasses eHealth-relevant research (for example, use of information and communications technologies for population health surveillance), the term was only recently introduced and has yet to contain any subcodes, limiting its usefulness at the present time. While the broader taxonomic categories each have their own character, there is clearly overlap between them. For example, decision support systems appear within both *Medical Informatics Applications* and *Medical Informatics Computing*, and electronic databases are a common feature in medical informatics applications, as well as representing a type of system.

Comparison of the MeSH tree with an expert-derived conceptual map endorsed by the International Medical Informatics Association (IMIA) revealed interesting differences in terms of the breadth of included concepts and their structural relationships (Table 2) [12]. For example, human and organizational factors appear to be underrepresented within Medline, while applications for consumers do not have a specific MeSH term (however, the IMIA taxonomy also appears to underrepresent consumer issues). This reflects the historical evolution of the MeSH hierarchy, which has been added to as the need arose by elaborating upon existing structures. Nonetheless, all the main areas apparently relevant to eHealth were encompassed by the MeSH tree and we are confident that using it as the basis of our search enabled the majority of pertinent literature to be identified.

**Figure 1:** Hierarchy of MeSH descriptors found below the Medical Informatics descriptor in the MeSH tree

**Table 2:** Medical informatics scientific content map endorsed by the International Medical Informatics Association (IMIA) [12]

**Applied Technology**	**Information Technology Infrastructure**	**Data-Infrastructure Related**	**Applications and Products**	**Human-Organizational**	**Education and Knowledge**
Algorithms Bioinformatics Biosignal processing Boolean logic Cryptology Human genome related Human interfaces Image processing Mathematical models in medicine Pattern recognition	Archival-repository systems for medical records- EPR-CPR-EMR Authentication Chip cards in health care Distributed systems Health professional workstation Interfaces Knowledge based systems Networks Neural networks Pen based Security Speech recognition Standards Systems architecture Telehealth User interfaces	Classification Coding systems Concept representation-preservation Data acquisition-data capture Data analysis-extraction tools Data entry Data policies Data protection Database design Indexing Syntax Language representation Lexicons Linguistics Modeling Nomenclatures Standards Terminology-vocabulary Thesaurus tools	Biostatistics Clinical trials Computer-supported surgery Decision support Diagnosis related Disease management EPR-CPR-EMR Epidemiological research Hosp IS Event-based systems Evidence based guidelines Expert systems Health services research Health Information Systems management Knowledge-based systems Laboratory data Image processing Operations/resource management Outcomes research and measurement Quality management Patient identification Patient monitoring Minimum data sets Supply chain Telematics Telemedicine	Assessment Compliance Cognitive tasks Collaboration Communication Economics of IT Ethics Implementation-deployment Diffusion of IT Evaluation Human Factors Legal issues, implementing national laws Management Managing change Needs assessment Organizational redesign processes Organizational transformation Planning Policy issues Privacy Project management Security Strategic plans Unique identifiers User-computer interface	Bibliographic Cognitive learning Computer aided instruction Computer-supported training Consumer education Continuing education Digital libraries E-Business Health/medical informatics education Information management- dissemination Knowledge bases Knowledge management Learning models Online/distance education
**Clinical Disciplines**: Anesthesia, Behavioral, Cardio/Thoracic, Cardiovascular, Dentistry, Dermatology, Emergency Medicine, Environmental Health, Gastroenterology, Human Genetics, Internal Medicine, Neurosurgery, Nursing, Obstetrics & Gynecology, Ophthalmology, Orthopedics, Pathology, Pediatrics, Pharmacy, Primary Care, Psychiatry, Radiology, Surgery, Urology					

### Exploring the Composition of the Medical Informatics Literature Using Existing Taxonomic Systems

Figure 2 describes trends in the volume and nature of the literature indexed by the *Medical Informatics* MeSH descriptor (note that searching for MeSH terms in PubMed automatically includes the more specific MeSH terms in a search). There has been a steady growth in the volume of medical informatics research literature. The annual number of publications increased from 1987 to 2003 five-fold.

**Figure 2:** Number of publications over time indexed with the MeSH descriptor Medical Informatics

Publications indexed with MeSH keywords from each of the 3 main medical informatics MeSH subtrees (*medical informatics computing, medical informatics applications, public health informatics*) all follow this steady upwards trend, as do most narrower MeSH (eg, *Information Systems; Therapy, Computer Assisted*). However, the frequency of publications concerned with *Clinical Laboratory Information* Systems (Figure 3), appears to be decreasing, while research concerned with computer-assisted diagnosis increased rapidly in 2003 (Figure 4).

A breakdown of Medical Informatics MeSH, including definition, year of introduction, number and type of publications is supplied in Multimedia Appendix 1.

**Figure 3:** Number of publications over time indexed with the MeSH descriptor Clinical Laboratory Information Systems

**Figure 4:** Number of publications over time indexed with the MeSH descriptor Diagnosis, Computer Assisted

### Using eHealth as a Search Term

As mentioned previously, there are currently no MeSH or equivalent coding categories in any of the databases searched which explicitly incorporate the term *eHealth* or its variants in their thesauri. This suggests that articles making reference to eHealth are being absorbed within existing classification schemes, such as Medline's *Medical Informatics* taxonomy.

When duplicates across databases were discarded we identified a total of 392 publications which explicitly referred to eHealth in the title, abstract, or journal title. Of these, most were represented in Medline. Appearing only in the Medline database were 283 (72%) articles, 54 (14%) only on the CINAHL database, and 55 (14%) only on the SCI, SSCI and ISTP databases.

**Figure 5:**
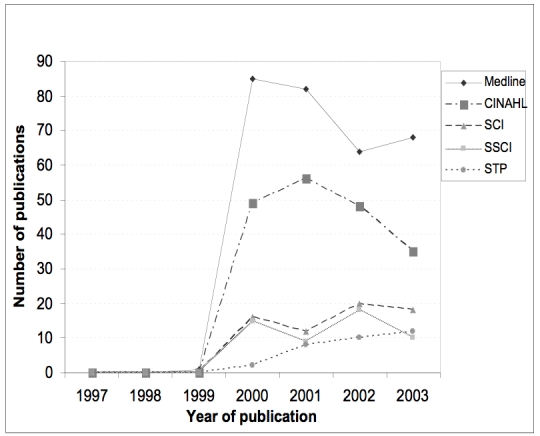
Number of publications found using the search term eHealth (or variants) in 5 research databases by year.

Figure 5 illustrates trends in the volume of eHealth publications appearing across databases over time. This shows that the term did not start to be used in the research literature until 2000. References to eHealth showed a dramatic rise in 2000 to 2001 and, despite a small dip in 2002 a general upward trend persists. Note that we also retrieved publications from the *Journal of Telemedicine and E-health* which were picked up due to the journal name, not necessarily because they dealt with eHealth.

### In Which Journals Do Publications Using the Term eHealth Appear?

In our study, publications containing the term *eHealth* were found in 154 different journals. A research fellow classified these by type, using a scheme agreed by the research team. The number of articles appearing within each journal were documented. Of the 387 publications found across multiple databases (after eliminating 5 that were clearly irrelevant), 77 appeared in clinical journals, 61 in health-services - related journals, 7 in finance-related journals, 4 in legal journals, 3 in journals related to medical education, and 28 in other journals not easily categorized. The journal titles with the most articles containing the term *eHealth* (n=9 for each journal) were the *Journal of Medical Internet Research*, *Managed Care Interface*, and *Journal of AHIMA / American Health Information Management Association*. The majority of publications were IT-related (207): however, among these, 116 articles were published in the *Journal of Telemedicine and E-health,* which were mainly picked up due to the journal name: only 4 articles actually contained the term *eHealth*in the abstract or title. Further details are provided in Table 3 and a detailed breakdown of journal titles is given in Multimedia Appendix 2.

**Table 3:** Topical areas of journal titles containing articles using the term eHealth

**Main Topic Area**	**More Specific Topics**	**Number of Publications (%)**
**Information Technology**	Telemedicine	124* (32%)
	Medical Informatics	35 (9%)
	Internet	23 (6%)
	Medical Computing	6 (1.5%)
	Biotechnology	2 (0.5%)
	Others	17 (4 %)
	**Sub total**	**207 (53%)**
**Clinical**	Specialist Medical	30 (8%)
	Generalist Medical	16 (4%)
	Nursing	13 (3%)
	Others	18 (4%)
	**Sub total**	**77 (19%)**
**Health Services**	Management	30 (8%)
	Case Management	16 (4%)
	Others	15 (4%)
	**Sub total**	**61 (16%)**
**Finance**	**Sub total**	**7 (2%)**
**Legal**	**Sub total**	**4 (1.5%)**
**Education**	**Sub total**	**3 (1.5%)**
**Others**	**Sub total**	**28 (7%)**
**Total**		**387 (100%)**

^*^ Of the 124 publications listed under telemedicine, 116 articles were published in the *Journal of Telemedicine and E-health*, of which only 4 articles actually contained the term *e-health*

### What Topics are Covered in the Literature Using the Term eHealth?

In our study, in order to identify the topics dealt with in papers explicitly referring to eHealth, article titles and abstracts were examined by a research fellow and classified using narrative descriptors. This indicated that the most common topics are related to telemedicine (25% of publications) or the Internet (13%), while some (6%) are concerned with issues such as the scope of eHealth, future trends, or progress and challenges in the field. Note that this view is possibly biased towards the telemedicine field, as all articles published in the *Journal of Telemedicine and E-health* were retrieved, even if they did not mention eHealth specifically. Other topics are distributed across a range of diffuse areas such as antiterrorism and medical errors, none of which is represented by more than 4 papers (hence relevant percentages have not been calculated). A heuristic summary is provided in Figure 6, which highlights the key topics and subtopics identified. These results are based on preliminary analysis; further validation work is underway. 

**Figure 6:** Map of topics in published articles using the term eHealth

### Definitions of eHealth

We identified 36 definitions of eHealth [13-52] appearing in published scientific abstracts and Web-based information sources (Table 4). As stated previously, our aim was not to perform an exhaustive and systematic review of definitions (which would have necessitated hand searching of full-text articles and reference lists), but to aggregate the most salient and easily accessible examples. Since many research databases are Internet accessible, there was some overlap between the definitions obtained by the two methods; however, they did yield largely unique results. In total, 36 definitions were identified. Definitions 1 to 15 were accessed via the research literature and 16 to 36 via the independent online searches, while 1, 5, 6, 7, 15 and 28 emerged from both searches.

Definitions were analyzed thematically in order to highlight specific technologies, applications or stakeholders referred to, and other theoretical concepts addressed, as detailed in Table 4. Analysis was initially performed by one investigator and the results checked by two others, thereby establishing agreement.

Our analysis suggests that there is significant variability in the scope and focus of existing definitions of eHealth both within the research literature and relevant sources on the World Wide Web. In terms of its functional scope, most definitions conceptualize eHealth as a broad range of medical informatics applications for facilitating the management and delivery of health care. Purported applications include dissemination of health-related information, storage and exchange of clinical data, interprofessional communication, computer-based support, patient-provider interaction and service delivery, education, health service management, health communities, and telemedicine, among others. A few narrow the concept down to specific applications, such as telemedicine or e-business, but these are the exceptions. While the range of applications is broad, a general theme relates to communication. One example is “E-health is connectivity; it is transactional; it is clinical. It is informational, interactive and interventional.”[43]

The majority of definitions (n=24) specify the use of networked information and communications technologies, primarily the Internet, and digital data, thus differentiating eHealth from the broader field of medical informatics, which incorporates “harder” technologies, such as scanning equipment, and bioinformatics research which tends to take place in isolation and is less directly applicable to health care service delivery. It is acknowledged that the Internet “…has the reach, the infrastructure, and the acceptance to achieve widespread change” [17] and it is envisaged that “Internet technology may rank with antibiotics, genetics and computers as among the most important changes for medical care delivery.”[16] Only 1 definition makes specific reference to harder technologies such as nanotechnology, robotics and laboratory tools [27], although another refers to Internet-compatible ICTs such as digital TV [40]. Of the 36 definitions identified, a sizable proportion make reference to telemedicine or telecare, either explicitly (7 examples) or in terms commonly used to describe these areas, such as delivery of care over distances. In most cases this is presented as part of a wider sphere of applications, although the definition from NHS Wales clearly identifies eHealth with telemedicine and telecare [45]. We identified 6 definitions that make explicit reference to business or e-business, although others contain related ideas such as the online trading of goods and services. In the majority of cases, such commercial applications are presented as merely one expression of eHealth.

In terms of the stakeholders considered to be the users or targets of eHealth, many definitions emphasize applications for providers and organizations–particularly those stressing electronic data exchange for clinical and administrative purposes. Others emphasize provision of information, education and services to consumers, including patients and “citizens”, with a small number clearly identifying eHealth with consumer health informatics [14, 46, 50]. Nevertheless the majority appear to encompass applications for *all stakeholder groups*, whether specified or implied by the breadth of the definition.

There is also variation in the degree to which alternative definitions consider wider theoretical issues, such as the influence of eHealth on society or on professional behaviour. Several highlight the changing cultural environment of health care; particularly growing patient empowerment (access to information and ability to use it), and point to the potential of eHealth to facilitate doctor-patient communication, partnership and shared decision making. Others emphasize the changes required to ensure that eHealth reaches its full potential, recognising that it requires new ways of working and attitudes and must take account of human and organizational influences affecting technology adoption and change. More broadly, eHealth is said to require a fundamental rethinking of health care processes and a commitment for networked global thinking to improve health care [22]. Overall, the definitions suggest a general excitement and optimism about the potential of this rapidly evolving field to improve health care processes and patient outcomes, and many clearly identify projected benefits such as improved clinical decision making, efficiency and safety.

**Table 4:** Definitions of eHealth identified from searching databases of scientific abstracts and wider Web-based information sources

**Definition**	**Source**	**Date**	**Technologies Specified**	**Applications Specified**	**Stakeholder Focus(and Other Concepts)**
1) “e-Health is a consumer-centred model of health care where stakeholders collaborate, utilizing ICTs, including Internet technologies to manage health, arrange, deliver and account for care, and manage the health care system”	Alvarez [13], based on Ontario Hospital e-health Council [14]	2002 (2002)	ICTs including Internet	General: manage health, arrange, deliver and account for care, and manage the health care system	Consumer centered but also emphasizes collaboration with providers
2) “Healthcare delivery is being transformed by advances in e-health and by the empowered, computer-literate public. Ready to become partners in their own health and to take advantage of online processes, health portals, and physician web pages and e-mail, this new breed of consumer is slowly redefining the physician/patient relationship. Such changes can effect positive results like improved clinical decision-making, increased efficiency, and strengthened communication between physicians and patients.”	Ball and Lillis [15]	2001	Internet online processes, health portals, physician en-pages, email.	General: healthcare delivery	Consumers (Change. Citizen empowerment. Physician/patient relationship/ communication. Improved clinical decision making, efficiency)
3) “The "e-health" era is nothing less than the digital transformation of the practice of medicine, as well as the business side of the health industry…. The Internet is the next frontier of health care. Health care consumers are flooding into cyberspace, and an Internet-based industry of health information providers is springing up to serve them. Internet technology may rank with antibiotics, genetics, and computers as among the most important changes for medical care delivery.”	Coile [16]	2000	Internet	The practice of medicine as well as the business side of the health industry	Consumers and providers (Change. New frontiers. Transformation of medical practice.)
4) “E-health—any electronic exchange of healthcare data or information across organizations—reflects an industry in transition…. The Internet clearly drives the development and adoption of e-health applications; standing alone, it has the reach, the infrastructure, and the acceptance to achieve widespread change.”	DeLuca and Enmark [17]	2000	Internet	Electronic exchange of healthcare data or information across organizations	Not specified. Implies focus on professional & organizational levels (Change)
5) "a new term needed to describe the combined use of electronic communication and information technology in the health sector... the use in the health sector of digital data - transmitted, stored and retrieved electronically - for clinical, educational and administrative purposes, both at the local site and at distance"	Della Mea [18], based on Mitchell [19]	2001 [1999]	Combined use of electronic communication in and IT in the health sector. Digital data transfer	Transmission of digital data locally and across distances, for clinical, educational and administrative purposes	Professionals and organizations
6) “e-health is the use of emerging information and communication technology, especially the Internet, to improve or enable health and healthcare.”	Eng [20], based on Eng [21]	2004 [2001]	Emerging ICTs, especially the Internet	General: To improve or enable health and health care	Not specified but implies consumers and providers
7) “e-health is an emerging field in the intersection of medical informatics, public health and business, referring to health services and information delivered or enhanced through the Internet and related technologies. In a broader sense, the term characterizes not only a technical development, but also a state-of-mind, a way of thinking, an attitude, and a commitment for networked, global thinking, to improve health care locally, regionally, and worldwide by using information and communication technology.”	Eysenbach [22]	2001	Broad definition encompassing many aspects of health informatics but focusing on the Internet and related technologies	Delivery of health services and information	Not specified. Implies consumers and providers. (“a state of mind, a way of thinking, an attitude and commitment for networked, global thinking to improve healthcare…”)
8) “Many of the major forces of change impacting health care today have technological underpinnings, and many of the less desirable impacts may have technological solutions. Two related technological forces are transacting business, online (e-business) and delivering health care online (e-health).”	Ellis and Schonfeld [23]	2001	Internet	General: Delivering healthcare	“Delivering” implies focus on professionals (Change. Relationship between eHealth and eBusiness)
9) “ehealth includes use of the internet or other electronic media to disseminate health related information or services.”	Gustafson and Wyatt [24]	2004	Internet or other electronic media	Dissemination of health related information or services	Implies consumers
10) “As a special expression of e-business in the health service the sphere of e-health has developed in recent years which increasingly manifests itself in the internet via health portals. Next to the transmitting of medical contents, the offer of community functions and the trading with goods from the medical sector, these health portals now increasingly provide advisory services for citizens by medical experts.”	Khorrami [25]	2002	Increasingly manifests itself in the Internet via health portals.	e-business Heath advice. Information exchange. Community functions. Advisory services for citizens	Consumers Healthcare organizations
11) “e-Health (use of interactive communication and information technologies to engage in health-related activities) includes not only telehealth-related media and telecommunications but also a wide array of consumer and healthcare provider activities that use the Internet.”	Maddox [26]	2002	Interactive ICT, telehealth, internet etc	General: health-related activities	Consumer and healthcare provider
12) “ …technologies with practical applications that have the potential to improve both quality of and access to healthcare….Telemedicine, Health Information Systems, Databases, Genomics, Biotechnology, eLearning, Continuing Professional Development, Nanotechnology, Drug Treatment Technologies, Decision Making Tools, Diagnostic Aids, eLibraries, Laboratory tools, and Robotics are all innovative or 'disruptive' technologies that promise a better health for our children.”	McConnell [27]	2002	Wide range of digital technologies	Wide range of informatics applications that may contribute to improved quality of and access to healthcare	Providers and patients (Quality. Access. “Disruptive technologies”)
13) “e-Health offers the rich potential of supplementing traditional delivery of services and channels of communication in ways that extend the healthcare organization's ability to meet the needs of its patients. Benefits include enhanced access to information and resources, empowerment of patients to make informed healthcare decisions, streamlined organizational processes and transactions, and improved quality, value, and patient satisfaction.”	Nazi [28]	2003	Not specified	Delivery of services Communication. Access to information and resources.	Patients (empowerment, satisfaction) Organization (efficiency and quality)
14) “the use of the Internet for health purposes”	Provost et al [29]	2003	Internet	General: “Health purposes”	Any
15) "a means of applying new low cost electronic technologies, such as 'web enabled' transactions, advanced networks and new design approaches, to healthcare delivery. In practice, it implies not only the application of new technologies, but also a fundamental re-thinking of healthcare processes based on using electronic communication and computer-based support at all levels and for all functions both within the healthcare service itself and in its dealings with outside suppliers. eHealth is a term which implies a way of working rather than a specific technology or application".	Richardson [30], based on Silicon Bridge [31]	2003 [2001]	Internet New low-cost electronic technologies such as ”web enabled” transactions and advanced networks”	General: “Healthcare delivery” Electronic communication and computer-based support at all levels and for all functions	‘Healthcare delivery [and] processes' implies organizational/ professional level (“…a way of working”)
16) “The healthcare industry's component of business over the internet.”	Blutt [32]	2001	Internet	Business	Implies organizations
17) "The application of the Internet and other related technologies in the healthcare industry to improve the access, efficiency, effectiveness, and quality of clinical and business processes utilized by healthcare organizations, practitioners, patients, and consumers to improve the health status of patients."	Broderick and Smaltz [33]	2003	Internet and related technologies	Improvement of access, efficiency, effectiveness and quality of clinical and business processes	Organizations, practitioners, patients, consumers
18) “eHealth includes the development, application and implementation of technology to improve effectiveness in healthcare. But it also includes getting it out there wherever it's needed in the service and making it happen across the service. It includes the use of telemedicine and clinical systems used for diagnosis and care pathways. We also apply the term to the policies and protocols that assure the confidentiality and security of sensitive data. Most of all it includes those aspects that support major change of working practice - training, support and Organisational Development.”	Chisholm [34]	2003	Technology	Telemedicine Clinical systems for diagnosis and care pathways Policies and protocols	Not specified, but implies organizational/professional focus (Importance of organizational and professional behaviour change recognized. Also confidentiality and security issues.)
19) “…using Information and Communications Technologies to ensure the right treatment to each patient, specialised to each individual's context and situation, and to deliver healthcare where patients and providers need not be in the same place at the same time.	CSIRO [35]	Un-dated	ICTs	Delivery of personalized patient care. Telemedicine implied	Not specified. Implies provider focus but also interaction with patients
20) "Put simply, e-health is a wide-ranging area of social policy that uses new media technologies to deliver both new and existing health outcomes. In the UK, it incorporates everything from NHS Direct online to Internet pharmacies to webcast operations involving consultants in another country…At the moment, the main focus of e-health is on patient empowerment and self-care. As the area develops, e-health could expand to include online long-term disease management, personalised health checks, and more efficient primary care services due to informed patients accessing the healthcare system at the most appropriate point."	GJW Government Relations Ltd [36]	2000	New media technologies	On-line health information Long-term disease management and patient self-care Telemedicine	Patients and professionals (Patients emphasized)
21) “something to do with computers, people, and health”(Centre for Global e-Health Innovation, 2003)	Gustafson [37]	2003	Computers in general	Very broad – computers, people and health	Implies all stakeholders
22) “the application of information and communication technologies (ICT) across the whole range of functions which, one way or another, affect the health of citizens and patients.”	European Commission [38]	2003	ICTs	Broad – the whole range of functions which, in one way or another, affect the health of citizens and patients	All stakeholders. Providers, patients, citizens.
23) “the emerging world of e-health can be defined as the application of information, communication and video technologies to the delivery of timely, professional and safe healthcare.”	European Health Telematics Association [39]	2004	ICT and video technologies	Broad – delivery of timely, professional and safe care	Not specified. Implies professional perspective.
24) “the use of emerging interactive technologies (i.e., Internet, interactive TV, interactive voice response systems, kiosks, personal digital assistants, CD-ROMs, DVD-ROMs) to enable health improvement and health care services. For this Initiative, these technologies should focus primarily on health behavior change and chronic disease management for consumers/patients.”	Health e-Technol-ogies Initiative [40]	2002	Emerging interactive technologies (Internet, interactive TV, interactive voice response systems, kiosks, personal digital assistants, CD-ROMs, DVD)	Enabling health improvement and health care services, chronic disease management, health behaviour change	Consumers, patients
25) “the use of ICT to support and improve healthcare”	Hoving et al [41]	2002	ICT	General: support and improve health care	Not specified.
26) "eHealth means taking the most recent developments in computer and networking technology, and applying it to the problems facing the healthcare community in all its forms - eHealth is the endeavour to produce reliable, easy-to-use, highly-automated, accurate systems, so that health care professionals can spend less time and resources on finalising the paperwork, and more time doing what they do best - taking care of people's health!"	IBA eHealth [42]	Un-dated	Recent developments in computer and networking technology	General: Applying it to the problems facing the healthcare community in all its forms Specific: administrative and clinical information to improve efficiency	Professionals (improved efficiency)
27) “The "e" is for electronic. Placed before the word health, it implies all things transmitted and technological in health care, which help improve the flow of information and the process of health care delivery. "E" networks integrate isolated towers of information and create new knowledge through the creation of relational databases. The spectrum of "E" is broad and goes beyond the use of a computer as a box on the desktop. It includes wireless communication using hand-held devices and the storage and function by the microchip which is revolutionizing health care, as it is inserted into everything we use to diagnose, treat, record, sort, analyze, and conclude. It also incorporates electronic forms of care delivery, such as telemedicine, providing health care over a distance, communicating by sound and image transmission. E-health is connectivity; it is transactional; it is clinical. It is informational, interactive and interventional.”	Marcus and Fabius [43]	Un-dated	Electronic networks, relational databases. Wireless communication.	All things transmitted and technological in health care, which help improve the flow of information and the process of health care delivery Electronic care delivery (telemedicine) Sound and image transmission	Not specified Connectivity; communication, interactivity, intervention
28) "the health services organisation and societal approach to health and health services which result from the introduction of, and increasing access to, new digital technologies: including the Internet, other computerised networks and tele- or distant health care facilitated by new digital technologies".	NHS SDO Programme [44]	2002	New digital technologies Internet Other computerized networks Telemedicine	Health service organization “Societal functions”	Organizations Society (citizens)
29) “More commonly known as “eHealth”, the headings of Telemedicine and Telecare are themselves subsumed under the framework category of "health informatics", which basically means the delivery of healthcare and medical knowledge through the application of advanced information and computer technologies.”	NHS Wales [45]	2003	Advanced information and computer technologies	Telemedicine and Telecare.	Not specified. (Identified eHealth with telemedicine)
30) “The big difference between yesterday's knowledge-based patient care and that of tomorrow is a fundamental premise that patients will explore the web world with a desire to learn more about their condition, including its treatment and prognosis. This has evolved into the concept of e-health”	Podichetty and Biscup [46]	2003	Internet	Patient information and decision support	Patients (Cultural shift to patient participation/ empowerment in health care)
31) “eHealth signifies a concerted effort undertaken by some leaders in healthcare and hi-tech industries to harness the benefits available through convergence of the internet and healthcare…”	Rx2000 Institute [47]	Un-dated	Internet	None specified	Not specified. Implies organizations (Harnessing benefits of converging internet and healthcare)
32) “eHealth describes the application of information and communications technologies (ICT) across the whole range of functions that help health. It is the means to deliver responsive healthcare tailored to the needs of the citizen.”	Silber [48]	2003	ICTs	Broad – the whole range of functions that help health	Citizens (consumers, patients, public)
33) “E-health is a new term used to describe the combined use of electronic communication and information technology in the health sector OR is the use, in the health sector, of digital data-transmitted, stored and retrieved electronically-for clinical, educational and administrative purposes, both at the local site and at a distance.”	WHO [49]	Un-dated	ICTs Digital data	Clinical, educational and administrative purposes, at the local site and at a distance	Organizations/professionals
34) “Using the internet and other electronic media to disseminate or provide access to health & lifestyle information or services”	Wyatt [50]	2003	Internet and other electronic media	Access to health and lifestyle information or services	Patients, public
35) “e-Health refers to all forms of electronic healthcare delivered over the Internet, ranging from informational, educational and commercial "products" to direct services offered by professionals, non-professionals, businesses or consumers themselves. e-Health includes a wide variety of the clinical activities that have traditionally characterized telehealth, but delivered through the Internet. Simply stated, e-Health is making health care more efficient, while allowing patients and professionals to do the previously impossible.”	Wysocki [51]	2001	Internet	Delivery of informational, educational and commercial "products" Direct delivery of services Clinical activities traditionally characterized telehealth	Professionals, consumers, businesses (Making health care more efficient, while allowing patients and professionals to do the previously impossible)
36) “E-health is a very broad term that encompasses many different activities related to the use of the Internet for healthcare. Many of these activities have focused on administrative functions such as claims processing or records storage. However, there is an increasing use of e-health related to patient and clinical care.”	American Telemed-icine Association [52]	2001	Internet	Administrative functions, patient and clinical care	Not specified. Implies organizational and professional focus (increasing use of eHealth for patient and clinical care)

## Discussion

We have established that *eHealth* is a new term which has yet to be formally represented in bibliographic research taxonomies but is part of the wider field of medical or health informatics. The *Medical Informatics* MeSH tree encompasses most topics likely to be classed as eHealth and is broadly compatible with an expert-derived taxonomy endorsed by IMIA. Since eHealth cuts across a range of health informatics topics a new MeSH term may neither be necessary nor appropriate at the present time. Topics related to eHealth are distributed across all component MeSH trees within the broader field, although most are represented by the *Medical Informatics Applications* tree, which emphasizes functions of technologies, rather than technologies themselves, and prioritizes delivery of clinical information, care or services. The medical informatics literature has grown steadily over the last 15 years although research on some topics, such as clinical laboratory information systems, is becoming less prevalent, while that on others, such as computer-assisted diagnosis, has recently increased rapidly, reflecting a change in emphasis from systems and database architectures to supportive applications.

Research articles explicitly referring to eHealth or its variants begun to appear in 2000 and are accumulating rapidly. The majority of such articles are indexed by Medline, although others appear in alternative databases. Such articles are published in a wide range of journals, spanning information science to law, but they are most commonly represented in journals related to health care information technology and telemedicine. A vast array of topics is covered by research articles referring to eHealth, highlighting the diffuse nature of the field and the lack of an agreed conceptual definition.

Definitions of eHealth demonstrate variation in the breadth and focus of alternative conceptualizations. At the extremes these range from the highly vague and diffuse, eg, “something to do with computers, people and health” [37] to the highly specific, eg, “the healthcare industry's component of business over the internet.” [32] Nevertheless, most conceptualize eHealth as a broad range of medical informatics applications for facilitating the management and delivery of health care, including dissemination of health-related information, storage and exchange of clinical data, interprofessional communication, computer-based support, patient-provider interaction, education, health service management, health communities and telemedicine, among other functions. A general theme relates to electronic communication, which is supported by the fact that most definitions specify the use of networked digital information and communications technologies, primarily the Internet. This differentiates eHealth from its parent field of medical informatics, which encompasses fixed technologies, such as X-Ray equipment, and pure bioinformatics research. While Internet technologies represent the prevailing theme, there is sufficient reference to applications that may be enabled by other interactive ICTs to suggest caution before identifying eHealth exclusively with this medium. This is supported by the high profile of decision support as a generic topic within the health informatics literature, which may, for example, take the form of clinical decision support systems or patient decision aids available via CD-ROM. Nevertheless, rapid increases in bandwidth and desktop computing capability make it likely that most such tools will soon be accessible using digital networked systems.

Many conceptualizations of eHealth incorporate telemedicine and although most do so as part of a wider sphere of applications, some authors use the terms synonymously [45]. We suggest that the latter is more likely due to a misuse of the term than, as some have speculated, “the death of telemedicine” in favour of eHealth [19] (cited in [18]). While telemedicine is certainly a theme in the eHealth literature, and the ICTs used in this area are common to many eHealth functions, it clearly represents only one domain of the broader field. Similarly, while several definitions extend to e-business, primarily meaning online transactions between suppliers and purchasers (2% of eHealth-related articles appear in journals of finance), most of these portray it as merely one application of eHealth for service management or care delivery.

Most definitions appear to encompass applications for all stakeholder groups, although many emphasize support for providers and organizations and a few see eHealth as an application of consumer health informatics or, even narrower, as the use of “internet and other electronic media to disseminate or provide access to health & lifestyle information or services.”[50] Our review of eHealth topics in the research and Web-based literature also indicates that the concept extends across stakeholder groups, including providers, patients, citizens, organizations, managers, academics and policymakers. A tendency has been noted for an inclusive model to predominate in Europe and a narrower consumer-focused one in the USA, possibly reflecting top-down versus bottom-up health systems and cultures [53]. However our results indicate that there is currently more overlap than difference between conceptualizations emanating from either side of the Atlantic, with the inclusive view predominating (also the case for Australia). Even of those conceptualizations tending toward the consumer informatics model, most emphasize interaction with professionals rather than simply passive delivery or provision of information to citizens or patients, thus drawing in the professional stakeholder. While there may be a valid argument for narrowing eHealth down to consumer health informatics in the future, namely to circumscribe the field and thereby make it more manageable, analysis of the existing eHealth landscape suggests that the concept is currently more inclusive.

Existing conceptualizations also vary in the extent to which they consider broader issues relating to the place, function or promise of eHealth in the modern world, such as its ability to promote patient self-care and communication, and the implications of this for the doctor-patient relationship. Many see eHealth as facilitating the transition of decision making control and responsibility from the professional to the empowered consumer, consistent with conceptions of the information age flipping over the “power pyramid” of health care [54]. The human and organizational changes required to effect new ways of working and attitudes also represent a strong theme. This is reflected in the relatively large number of publications, identified by the keyword search, that are concerned with issues such as challenges to implementation, as opposed to specific technologies or applications. We therefore agree that the concept incorporates “a state-of-mind, a way of thinking, an attitude.” [22] Such human and organizational factors appear to be underrepresented in the MeSH *Medical Informatics* taxonomy at present, suggesting that a review may be warranted to bring it into line with expert-derived ontologies such as that endorsed by IMIA. More broadly, eHealth is said to require a fundamental rethinking of healthcare processes” [31] and a “commitment for networked global thinking to improve healthcare” [22], but there is clearly a general optimism surrounding the potential benefits of this rapidly evolving field for health care processes and patient outcomes.

Of course, definitions do not exist in isolation and the source documents for those reviewed provide further elaboration. For example, Eng provides a “5 C's model” of functions and capabilities of eHealth (content, connectivity, community, commerce, care) [21]; Eysenbach lists “10 essential E's” in eHealth (efficiency, enhancing quality of care, evidence-based, empowerment of consumers, etc) [22], and Richardson proposes a “4-pillar model” (under the headings of clinical applications, healthcare professional continuing education, public health information, and education and lifetime health plan) [30]. Yet others have attempted to define eHealth in terms of its potential role during a patient's care pathway [55] or with reference to the settings in which it may be useful [48]. Nonetheless, most authors have successfully distilled their concepts within the definitions they provide. Converging these with the other information sources documented in this report provides a fairly comprehensive overview of the concept and enables us to draw broad conclusions about its nature and scope.

In an editorial on the website, Health Informatics Europe, Ahmad Risk posed the question: “So, is this it? … Does 'eHealth' mean 'web health informatics'?”[9] Based on our results, our conclusion is largely “Yes”, or “It soon will be”, recognising that the parameters of the field currently extend to other interactive ICTs which, with increasing computing power, bandwidth and wireless capability, may rapidly be accommodated by Internet technologies. Based on our analysis of the place of eHealth within the wider informatics field and the nature of research activity and general commentary on the topic, we conclude that it is well represented by the global definitions suggested by Eng and Eysenbach early in the emergence of the field, with a minor change to the latter, as indicated below:

e-health is the use of emerging information and communications technology, especially the Internet, to improve or enable health and healthcare. [21]

e-health is an emerging field of medical informatics, referring to the organization and delivery of health services and information using the Internet and related technologies. In a broader sense, the term characterizes not only a technical development, but also a new way of working, an attitude, and a commitment for networked, global thinking, to improve health care locally, regionally, and worldwide by using information and communication technology. (adapted from Eysenbach [22])

## Multimedia Appendix 1

Medline MeSH descriptors potentially relevant to eHealth.

## Multimedia Appendix 2

Journals in which publications explicitly the terms *eHealth*, *e-health*, or *e health* appear.

## Abbreviations

ICT: Information and Communications Technology
IMIA: International Medical Informatics Association
IT: Information Technology
MeSH: Medical Subject Headings
NHS: National Health Service
