# Supplementary material for: What Is eHealth (4): A Scoping Exercise to Map the Field
Source: J Med Internet Res. 2005 Mar 31;7(1):e9. doi: 10.2196/jmir.7.1.e9 (PMC1550637; doi:10.2196/jmir.7.1.e9)
Supplement: Supplementary file 1 [file jmir_v7i1e9_app1.doc]

Appendix 1

**Medline MeSH potentially relevant to eHealth (Medical Informatics and below),**

**Including definitions, year of introduction and breakdown of content.** [Results to June 2004.]

| **MeSH (level)** | **Definition** | **Year introd-uced** | **Number of public-ations** | **Reviews** | **CT/**  **RCT** | **Meta-analyses** | **"Review, Academic" [Publication Type]** |
| --- | --- | --- | --- | --- | --- | --- | --- |
| **Medical Informatics (1)** | The field of information science concerned with the analysis and dissemination of medical data through the application of computers to various aspects of health care and medicine. | 1987 | 320134 | 23458 | 9342 | 554 | 1447 |
| **Medical Informatics Applications (1.1)** | Automated systems applied to the patient care process including diagnosis, therapy, and systems of communicating medical data within the health care setting. | 1987 | 91862 | 6740 | 2048 | 332 | 507 |
| **Decision Making, Computer-Assisted (1.1.1)** | Use of an interactive computer system designed to assist the physician or other health professional in choosing between certain relationships or variables for the purpose of making a diagnostic or therapeutic decision. | 1987 | 24295 | 1903 | 1363 | 16 | 109 |
| **Diagnosis, Computer-Assisted (1.1.1.1)** | Application of computer programs designed to assist the physician in solving a diagnostic problem. | 1991 | 14272 | 934 | 723 | 6 | 38 |
| **Image Interpretation, Computer-Assisted (1.1.1.1.1)** | Computer systems developed to aid in the interpretation of ultrasound, radiographic images, etc. | 1987 | 182374 | 20158 | 5802 | 121 | 906 |
| **Therapy, Computer-Assisted (1.1.1.2)** | Computer systems utilized as adjuncts in the treatment of disease. | 1987 | 9244 | 878 | 598 | 9 | 62 |
| **Drug Therapy, Computer-Assisted (1.1.1.2.1)** | Adjunctive computer programs in providing drug treatment to patients. | 1991 | 593 | 38 | 63 | 5 | 4 |
| **Radiotherapy, Computer-Assisted (1.1.1.2.2)** | Computer systems or programs used in accurate computations for providing radiation dosage treatment to patients. | 1987 | 5799 | 526 | 301 | 2 | 44 |
| **Surgery, Computer-Assisted (1.1.1.2.3)** | Surgical procedures conducted with the aid of computers. This is most frequently used in orthopedic and laproscopic surgery for implant placement and instrument guidance. Image-guided surgery interactively combines prior CT scans or MRI images with real-time video. | 2002 | 792 | 99 | 49 | 0 | 8 |
| **Information Storage and Retrieval (1.1.2)** | A branch of computer or library science relating to the storage, locating, searching, and selecting, upon demand, relevant data on a given subject. | 1991 | 7026 |  |  |  |  |
| **Information Systems (1.1.3)** | Integrated set of files, procedures, and equipment for the storage, manipulation, and retrieval of information. | 1982 | 67818 | 4836 | 719 | 316 | 389 |
| **Clinical Laboratory Information Systems (1.1.3.1)** | Information systems, usually computer-assisted, designed to store, manipulate, and retrieve information for planning, organizing, directing, and controlling administrative and clinical activities associated with the provision and utilization of clinical laboratory services. | 1991 | 1307 | 100 | 2 | 0 | 2 |
| **Community Networks (1.1.3.2)** | Organizations and individuals cooperating together toward a common goal at the local or grassroots level. | 1996 | 1828 | 123 | 23 | 2 | 4 |
| **Databases (1.1.3.3)** | Organized collections of computer records, standardized in format and content, that are stored in any of a variety of computer-readable modes. They are the basic sets of data from which computer-readable files are created. (from ALA Glossary of Library and Information Science, 1983) | 1998 | 26167 | 2986 | 185 | 285 | 316 |
| **Decision Support Systems, Clinical (1.1.3.4)** | Computer-based information systems used to integrate clinical and patient information and provide support for decision-making in patient care. | 1998 | 1090 | 81 | 32 | 5 | 4 |
| **Geographic Information Systems (1.1.3.5)** | Computer systems capable of assembling, storing, manipulating, and displaying geographically referenced information, i.e. data identified according to their locations. | 2003 | 269 | 23 | 0 | 0 | 4 |
| **Hospital Information Systems (1.1.3.6)** | Integrated, computer-assisted systems designed to store, manipulate, and retrieve information concerned with the administrative and clinical aspects of providing medical services within the hospital. | 1987 | 13340 | 730 | 191 | 3 | 24 |
| **Integrated Advanced Information Management Systems (1.1.3.7)** | A concept, developed in 1983 under the aegis of and supported by the National Library of Medicine under the name of Integrated Academic Information Management Systems, to provide professionals in academic health sciences centers and health sciences institutions with convenient access to an integrated and comprehensive network of knowledge. It addresses a wide cross-section of users from administrators and faculty to students and clinicians and has applications to planning, clinical and managerial decision-making, teaching, and research. It provides access to various types of clinical, management, educational, etc., databases, as well as to research and bibliographic databases. In August 1992 the name was changed from Integrated Academic Information Management Systems to Integrated Advanced Information Management Systems to reflect use beyond the academic milieu. | 1994 | 232 | 14 | 0 | 0 | 0 |
| **Management Information Systems (1.1.3.8)** | Systems designed to provide information primarily concerned with the administrative functions associated with the provision and utilization of services; also includes program planning, etc. | 1987 | 20760 | 1028 | 238 | 4 | 33 |
| **Ambulatory Care Information Systems (1.1.3.8.1)** | Information systems, usually computer-assisted, designed to store, manipulate, and retrieve information for planning, organizing, directing, and controlling administrative activities associated with the provision and utilization of ambulatory care services and facilities. | 1991 | 761 | 17 | 12 | 0 | 2 |
| **Clinical Pharmacy Information Systems (1.1.3.8.2)** | Information systems, usually computer-assisted, designed to store, manipulate, and retrieve information for planning, organizing, directing, and controlling administrative activities associated with the provision and utilization of clinical pharmacy services. | 1991 | 613 | 22 | 7 | 0 | 1 |
| **Database Management Systems (1.1.3.8.3)** | Software designed to store, manipulate, manage, and control data for specific uses. | 1991 | 2999 | 143 | 14 | 1 | 5 |
| **Decision Support Systems, Management (1.1.3.8.4)** | Computer-based systems that enable management to interrogate the computer on an ad hoc basis for various kinds of information in the organization, which predict the effect of potential decisions. | 1991 | 668 | 26 | 3 | 0 | 0 |
| **Office Automation (1.1.3.8.5)** | Use of computers or computer systems for doing routine clerical work, e.g., billing, records pertaining to the administration of the office, etc. | 1987 | 991 | 38 | 15 | 0 | 0 |
| **Word Processing (1.1.3.8.5.1)** | Automated production of typewritten documents with text editing and storage functions using computer software. | 1989 | 359 | 11 | 10 | 0 | 0 |
| **Personnel Staffing and Scheduling Information Systems (1.1.3.8.6)** | Computer-based systems for use in personnel management in a facility, e.g., distribution of caregivers with relation to patient needs. | 1991 | 342 | 6 | 1 | 0 | 0 |
| **Radiology Information Systems (1.1.3.8.7)** | Information systems, usually computer-assisted, designed to store, manipulate, and retrieve information for planning, organizing, directing, and controlling administrative activities associated with the provision and utilization of radiology services and facilities. | 1991 | 3216 | 257 | 25 | 2 | 10 |
| **Teleradiology (1.1.3.8.7.1)** | The electronic transmission of radiological images from one location to another for the purposes of interpretation and/or consultation. Users in different locations may simultaneously view images with greater access to secondary consultations and improved continuing education. (From American College of Radiology, ACR Standard for Teleradiology, 1994, p3) | 1996 | 728 | 79 | 11 | 0 | 4 |
| **Operating Room Information Systems (1.1.3.6.1)** | Information systems, usually computer-assisted, designed to store, manipulate, and retrieve information for planning, organizing, directing, and controlling administrative activities associated with the provision and utilization of operating room services and facilities. | 1991 | 344 | 17 | 1 | 0 | 0 |
| **Point-of-Care Systems (1.1.3.6.2)** | Laboratory and other services provided to patients at the bedside. These include diagnostic and laboratory testing using automated information entry systems. | 1996 | 1901 | 237 | 132 | 0 | 10 |
| **Medical Informatics Computing (1.2)** | Precise procedural mathematical and logical operations utilized in the study of medical information pertaining to health care. | 1987 | 268858 | 19133 | 7957 | 245 | 1056 |
| **Computer Literacy (1.2.1)** | General learning, knowledge, and fluency with computer terms; also, becoming familiar with how computers operate and how they are programmed. | 1987 | 687 | 32 | 15 | 0 | 0 |
| **Computer Systems (1.2.2)** | Systems composed of a computer or computers, peripheral equipment, such as disks, printers, and terminals, and telecommunications capabilities. | 1987 | 84715 | 3582 | 1025 | 12 | 111 |
| **Computer Communication Networks (1.2.2.1)** | A system containing any combination of computers, computer terminals, printers, audio or visual display devices, or telephones interconnected by telecommunications equipment or cables: used to transmit or receive information. (Random House Unabridged Dictionary, 2d ed) | 1991 | 23368 | 1681 | 168 | 6 | 57 |
| **Internet (1.2.2.1.1)** | A loose confederation of computer communication networks around the world. The networks that make up the Internet are connected through several backbone networks. The Internet grew out of the US Government ARPAnet project and was designed to facilitate information exchange. | 1999 | 13922 | 1151 | 123 | 5 | 44 |
| **Local Area Networks (1.2.2.1.2)** | Communications networks connecting various hardware devices together within or between buildings by means of a continuous cable or voice data telephone system. | 1991 | 609 | 30 | 3 | 0 | 0 |
| **Computers (1.2.2.2)** | No definition given | 1966 | 58102 | 1758 | 801 | 6 | 46 |
| **Computer Peripherals (1.2.2.2.1)** | Various units or machines that operate in combination or in conjunction with a computer but are not physically part of it. Peripheral devices typically display computer data, store data from the computer and return the data to the computer on demand, prepare data for human use, or acquire data from a source and convert it to a form usable by a computer. (Computer Dictionary, 4th ed.) | 1991 | 2791 | 189 | 119 | 2 | 6 |
| **Computer Storage Devices (1.2.2.2.1.1)** | Devices capable of receiving data, retaining data for an indefinite or finite period of time, and supplying data upon demand. | 1991 | 1200 | 70 | 29 | 1 | 2 |
| **Optical Storage Devices (1.2.2.2.1.1.1)** | A computer disk read by a laser beam, containing data prerecorded by a vendor. The buyer cannot enter or modify data in any way but the advantages lie in the speed of accessibility, relative immunity to damage, and relatively low cost of purchase. | 1991 | 1393 | 59 | 45 | 1 | 2 |
| **Computer Terminals (1.2.2.2.1.2)** | Input/output devices designed to receive data in an environment associated with the job to be performed, and capable of transmitting entries to, and obtaining output from, the system of which it is a part. (Computer Dictionary, 4th ed.) | 1991 | 981 | 93 | 73 | 1 | 4 |
| **Modems (1.2.2.2.1.3)** | Equipment that sends digital information over telephone lines. The term Modem is a short form of the phrase modulator-demodulator. | 2000 | 211 | 9 | 6 | 0 | 0 |
| **Computers, Molecular (1.2.2.2.2)** | Computers whose input, output and state transitions are carried out by biochemical interactions and reactions. | 2003 | 49 | 9 | 0 | 0 | 1 |
| **Microcomputers (1.2.2.2.3)** | Small computers using LSI (large-scale integration) microprocessor chips as the CPU (central processing unit) and semiconductor memories for compact, inexpensive storage of program instructions and data. They are smaller and less expensive than minicomputers and are usually built into a dedicated system where they are optimized for a particular application. "Microprocessor" may refer to just the CPU or the entire microcomputer. | 1989 | 12698 | 380 | 240 | 1 | 6 |
| **Computers, Handheld (1.2.2.2.3.1)** | MICROCOMPUTERS, sometimes called PDA, that are very small and portable, fitting in a hand, and that have much more function than a calculator. They are convenient to use in clinical and other field situations for quick data management. They usually require docking with MICROCOMPUTERS for updates. | 2003 | 268 | 20 | 5 | 0 | 2 |
| **Minicomputers (1.2.2.2.4)** | Small computers that lack the speed, memory capacity, and instructional capability of the full-size computer but usually retain its programmable flexibility. They are larger, faster, and more flexible, powerful, and expensive than microcomputers. | 1991 | 935 | 20 | 4 | 0 | 0 |
| **Computing Methodologies (1.2.3)** | Computer-assisted analysis and processing of problems in a particular area. | 1997 | 175012 | 15243 | 6525 | 226 | 919 |
| **Artificial Intelligence (1.2.3.1)** | The study and implementation of techniques and methods for designing computer systems to perform functions normally associated with human intelligence, such as understanding language, learning, reasoning, problem solving, etc. | 1986 | 13569 | 1295 | 282 | 3 | 81 |
| **Expert Systems (1.2.3.1.1)** | Computer programs based on knowledge developed from consultation with experts on a problem, and the processing and/or formalizing of this knowledge using these programs in such a manner that the problems may be solved. | 1987 | 2322 | 231 | 41 | 1 | 6 |
| **Robotics (1.2.3.1.2)** | The application of electronic, computerized control systems to mechanical devices designed to perform human functions. Formerly restricted to industry, but nowadays applied to artificial organs controlled by bionic (bioelectronic) devices, like automated insulin pumps and other prostheses. | 1987 | 2321 | 286 | 74 | 0 | 17 |
| **Public Health Informatics**  **(1.3)** | The systematic application of information and computer sciences to public health practice, research, and learning. It is the discipline that integrates public health with information technology. The development of this field and dissemination of informatics knowledge and expertise to public health professionals is the key to unlocking the potential of information systems to improve the health of the nation. ([www.nlm.nih.gov/pubs/cbm/phi2001.html](http://www.nlm.nih.gov/pubs/cbm/phi2001.html)) | 2003 | 219 | 18 | 0 | 0 | 5 |
| **Communications Media (2)** | The means of interchanging or transmitting and receiving information. Historically the media were written: books, journals, newspapers, and other publications; in the modern age the media include, in addition, radio, television, computers, and information networks. | 1998 | 122726 | 5306 | 2504 | 74 | 378 |
| **Telecommunications (2.1)** | Transmission of information over distances via electronic means. | 1976 | 25165 | 1186 | 774 | 10 | 67 |
| **Electronic Mail (2.1.1)** | Messages between computer users via COMPUTER COMMUNICATION NETWORKS. This feature duplicates most of the features of paper mail, such as forwarding, multiple copies, and attachments of images and other file types, but with a speed advantage. The term also refers to an individual message sent in this way. | 2003 | 254 | 10 | 5 | 0 | 0 |
| **Radio (2.1.2)** | The transmission and reception of electric impulses or signals by means of electric waves without a connecting wire, or the use of these waves for the wireless transmission of electric impulses into which sound is converted. (From Webster's 3d) | ? | 1491 | 16 | 14 | 0 | 0 |
| **Satellite Communications (2.1.3)** | Communications using an active or passive satellite to extend the range of radio, television, or other electronic transmission by returning signals to earth from an orbiting satellite. | 1993 | 293 | 40 | 3 | 0 | 1 |
| **Telefacsimile (2.1.4)** | A telecommunication system combining the transmission of a document scanned at a transmitter, its reconstruction at a receiving station, and its duplication there by a copier. | 1992 | 151 | 4 | 0 | 0 | 0 |
| **Telemedicine (2.1.5)** | Delivery of health services via remote telecommunications. This includes interactive consultative and diagnostic services. | 1993 | 6059 | 572 | 197 | 3 | 42 |
| **Remote Consultation (2.1.5.1)** | Consultation via remote telecommunications, generally for the purpose of diagnosis or treatment of a patient at a site remote from the patient or primary physician. | 1996 | 1694 | 97 | 85 | 0 | 6 |
| **Telepathology (2.1.5.2)** | Transmission and interpretation of tissue specimens via remote telecommunication, generally for the purpose of diagnosis or consultation but may also be used for continuing education. | 1996 | 370 | 56 | 11 | 1 | 3 |
| **Telephone (2.1.6)** | An instrument for reproducing sounds especially articulate speech at a distance. (Webster, 3rd ed) | 1974 | 5912 | 165 | 448 | 6 | 8 |
| **Answering Services (2.1.6.1)** | Communication services provided by a person or a machine to record and relay the message from the caller. | 2003 | 8 | 0 | 1 | 0 | 0 |
| **Cellular Phone (2.1.6.2)** | Analog or digital communications device in which the user has a wireless connection from a telephone to a nearby transmitter. It is termed cellular because the service area is divided into multiple "cells." As the user moves from one cell area to another, the call is transferred to the local transmitter. | 2003 | 210 | 13 | 11 | 0 | 0 |
| **Television (2.1.7)** | The transmission and reproduction of transient images of fixed or moving objects. An electronic system of transmitting such images together with sound over a wire or through space by apparatus that converts light and sound into electrical waves and reconverts them into visible light rays and audible sound. (From Webster, 3rd ed) | ? | 17807 | 553 | 704 | 5 | 20 |
| **Microscopy, Video (2.1.7.1)** | Microscopy in which television cameras are used to brighten magnified images that are otherwise too dark to be seen with the naked eye. It is used frequently in TELEPATHOLOGY. | 1995 | 2493 | 140 | 18 | 0 | 7 |
| **Communication (3)** | The exchange or transmission of ideas, attitudes, or beliefs between individuals or groups. | ? | 187885 | 14344 | 4488 | 121 | 844 |
| **Cybernetics (3.1)** | That branch of learning which brings together theories and studies on communication and control in living organisms and machines. | ? | 19402 | 2208 | 729 | 6 | 192 |
| **Feedback (3.1.1)** | A mechanism of communication within a system in that the input signal generates an output response which returns to influence the continued activity or productivity of that system. | 1965 | 18007 | 2120 | 724 | 6 | 191 |
| **Bionics**  **(belongs in biophysics tree of MeSH)** | The study of systems, particularly electronic systems, which function after the manner of, in a manner characteristic of, or resembling living systems. Also, the science of applying biological techniques and principles to the design of electronic systems. | 1970 | 256 | 24 | 1 | 0 | 0 |
| **Diffusion of Innovation (3.2)** | The broad dissemination of new ideas, procedures, techniques, materials, and devices and the degree to which these are accepted and used. | 1991 | 5386 | 643 | 48 | 4 | 19 |
| **Technology Transfer (3.2.1)** | Spread and adoption of inventions and techniques from one geographic area to another, from one discipline to another, or from one sector of the economy to another. For example, improvements in medical equipment may be transferred from industrial countries to developing countries, advances arising from aerospace engineering may be applied to equipment for persons with disabilities, and innovations in science arising from government research are made available to private enterprise. | 1995 | 840 | 94 | 8 | 0 | 2 |
| **Hotlines (3.3)** | A direct communication system, usually telephone, established for instant contact. It is designed to provide special information and assistance through trained personnel and is used for counseling, referrals, and emergencies such as poisonings and threatened suicides. | 1989 | 1189 | 70 | 30 | 2 | 5 |
| **Interdisciplinary Communication (3.4)** | Communication, in the sense of cross-fertilization of ideas, involving two or more academic disciplines (such as the disciplines that comprise the cross-disciplinary field of bioethics, including the health and biological sciences, the humanities, and the social sciences and law). Also includes problems in communication stemming from differences in patterns of language usage in different academic or medical disciplines. | 2003 | 1339 | 126 | 4 | 0 | 8 |
| **Computer Security (4)** | Protective measures against unauthorized access to or interference with computer operating systems, telecommunications, or data structures, especially the modification, deletion, destruction, or release of data in computers. It includes methods of forestalling interference by computer viruses or so-called computer hackers aiming to compromise stored data. | 1992 | 2569 | 116 | 6 | 0 | 3 |
| **Information Management (5)** | Management of the acquisition, organization, storage, retrieval, and dissemination of information. (From Thesaurus of ERIC Descriptors, 1994) | 1997 | 1502 | 64 | 4 | 1 | 4 |
