# Supplementary material for: What Is eHealth (4): A Scoping Exercise to Map the Field
Source: J Med Internet Res. 2005 Mar 31;7(1):e9. doi: 10.2196/jmir.7.1.e9 (PMC1550637; doi:10.2196/jmir.7.1.e9)
Supplement: Supplementary file 2 [file jmir_v7i1e9_app2.doc]

Appendix 2

Journals in which publications explicitly containing the terms ‘eHealth’, ‘e-health’, or ‘e health’ appear

| **Journal** | **Number of articles** |
| --- | --- |
| **Clinical Journals** | |
| Acta Haematol | 1 |
| ALLERGY | 1 |
| Am J Health Behav | 1 |
| AM J KIDNEY DIS | 1 |
| Am J Med Qual | 1 |
| Am J Prev Med | 1 |
| Ann Chir Plast Esthet | 1 |
| Arizona Nurse | 1 |
| BASIC APPL SOC PSYCH | 1 |
| BRIT MED J | 4 |
| Bull Cancer | 1 |
| CAN MED ASSOC J | 1 |
| Caring | 2 |
| Clin Dermatol | 2 |
| CONTEMP PSYCHOL | 1 |
| CONTROL CLIN TRIALS | 1 |
| CRIT CARE MED | 2 |
| Dis Manag Advis | 1 |
| Family Practice Management | 2 |
| Hematol Oncol Clin North Am | 1 |
| Hosp Pract (Off Ed) | 1 |
| INT J GERIATR PSYCH | 1 |
| INT J STRESS MANAGE | 1 |
| International Journal of Language & Communication Disorders | 1 |
| IRISH J MED SCI | 1 |
| J Am Diet Assoc | 1 |
| J Cardiovasc Manag | 1 |
| J HEALTH PSYCHOL | 3 |
| J Invasive Cardiol | 1 |
| J INVEST MED | 1 |
| J Midwifery Womens Health | 1 |
| J Oncol Manag | 1 |
| J ORG COMP ELECT COM | 1 |
| J RHEUMATOL | 1 |
| J VOCAT BEHAV | 1 |
| Jama | 2 |
| Journal of Manual and Manipulative Therapy | 1 |
| Lippincott's Case Management | 1 |
| Med Lett Health After 50 | 1 |
| Missouri Nurse | 1 |
| Mod Healthc | 4 |
| NURS ECON | 1 |
| Nurse Pract | 1 |
| Nursing Spectrum | 1 |
| Nursing Times | 2 |
| Online Journal of Issues in Nursing | 1 |
| ORL Head Neck Nurs | 1 |
| P ASIST ANNU | 1 |
| Patient Care Manag | 1 |
| PHARM WORLD SCI | 1 |
| Physician Exec | 5 |
| Policy, Politics, & Nursing Practice | 1 |
| PROF PSYCHOL-RES | 1 |
| RADIOLOGE | 1 |
| Rehabil Nurs | 1 |
| Semin Nurse Manag | 1 |
| Singapore Med J | 1 |
| Spine | 1 |
| Trop Doct | 1 |
| **Sub Total (%)** | **77 (20%)** |
|  | |
| **Education Journals** | |
| Nurs Educ Perspect | 1 |
| Journal of Nursing Education | 1 |
| International Electronic Journal of Health Education | 1 |
| **Sub Total (%)** | **3 (1%)** |
|  | |
| **Finance Journals** | |
| Business & Health | 3 |
| Patient Acc | 1 |
| Healthc Financ Manage | 2 |
| Med Econ | 1 |
| **Sub Total (%)** | **7 (2%)** |
|  | |
| **Legal Journals** | |
| Am J Law Med | 1 |
| J Health Law | 2 |
| J Leg Med | 1 |
| **Sub Total (%)** | **4 (1%)** |
|  | |
| **Health Services Related Journals** | |
| Case Management | 1 |
| Case Management Advisor | 3 |
| Case Manager | 3 |
| Clin Leadersh Manag Rev | 1 |
| Front Health Serv Manage | 4 |
| Health Care Manager | 2 |
| Health Care Strateg Manage | 1 |
| Health Forum J | 5 |
| Health Service Journal | 1 |
| Healthc Leadersh Manag Rep | 1 |
| Healthplan | 1 |
| Hosp Case Manag | 1 |
| Hospitals & Health Networks | 5 |
| Hospital Quarterly | 2 |
| Issue brief (Grantmakers Health) | 1 |
| J Health Commun | 1 |
| J Health Serv Res Policy | 1 |
| J Healthc Inf Manag | 5 |
| J Med Pract Manage | 1 |
| Manag Care | 1 |
| Manag Care Interface | 9 |
| Manag Care Q | 3 |
| Mark Health Serv | 3 |
| Med Group Manage J | 1 |
| Mich Health Hosp | 2 |
| Qual Lett Healthc Lead | 1 |
| World Hosp Health Serv | 1 |
| **Sub Total (%)** | **61 (16%)** |
|  | |
| **Information technology Journals** | |
| AHIMA Advantage | 1 |
| AI COMMUN | 1 |
| Am J Health Syst Pharm | 1 |
| AUSTRALAS BIOTECHNOL | 1 |
| Biomed Instrum Technol | 1 |
| COMPUT BIOL MED | 1 |
| COMPUT COMMUN | 1 |
| CYBERPSYCHOL BEHAV | 1 |
| EXPERT SYST APPL | 1 |
| Health Data Manag | 5 |
| Health Information Management Journal | 2 |
| Health Management Technology | 4 |
| Healthcare Informatics | 3 |
| Ihrim | 1 |
| INFORM SYST | 1 |
| Information World Review | 1 |
| Int J Med Inf | 4 |
| Internet Healthc Strateg | 4 |
| Internet Medicine | 1 |
| J Ahima | 9 |
| J Am Med Inform Assoc | 1 |
| J COMPUT INFORM SYST | 1 |
| J Med Internet Res | 9 |
| J MED SYST | 3 |
| J Med Syst | 1 |
| J Telemed Telecare | 6 |
| J UNIVERS COMPUT SCI | 1 |
| Journal of the Medical Library Association | 2 |
| MD Comput | 1 |
| MED INFORM INTERNET | 1 |
| Medical Information Technology Law Report | 1 |
| Medicine on the Net | 8 |
| Medinfo | 4 |
| METHOD INFORM MED | 2 |
| On-Line Journal of Nursing Informatics | 1 |
| SOC SCI COMPUT REV | 1 |
| TECHNOL REV | 1 |
| Telemed J E Health | 116 |
| Telemed Today | 2 |
| Topics in Health Information Management | 1 |
| **Sub Total (%)** | **207 (53%)** |
|  | |
| **Others** | |
| ACSMS HEALTH FIT | 1 |
| AVIAT SPACE ENVIR MD | 1 |
| CurtinCalls | 1 |
| GESUNDHEITSWESEN | 1 |
| Health Aff (Millwood) | 8 |
| Health Estate | 1 |
| Healthweek (Texas) | 1 |
| J INST BRIT TELECOMM | 1 |
| J SCI IND RES INDIA | 1 |
| Khirurgiia (Mosk) | 1 |
| Los Angeles Times | 1 |
| Perspectives | 1 |
| QRC Advis | 1 |
| Russ Coiles Health Trends | 1 |
| Soc Sci Med | 1 |
| SCI SOC SANTE | 1 |
| Trustee | 3 |
| Value Health | 2 |
| **Sub Total (%)** | **28 (7%)** |
|  |  |
| **Grand Total** | **387** |
